# An Appraisal of the Oleocanthal-Rich Extra Virgin Olive Oil (EVOO) and Its Potential Anticancer and Neuroprotective Properties

**DOI:** 10.3390/ijms242417323

**Published:** 2023-12-10

**Authors:** Raffaele Infante, Marco Infante, Donatella Pastore, Francesca Pacifici, Francesca Chiereghin, Gina Malatesta, Giulia Donadel, Manfredi Tesauro, David Della-Morte

**Affiliations:** 1 Department of Human Sciences and Quality of Life Promotion, San Raffaele University, 00166 Rome, Italy; raffaeleinfante.pharmd@gmail.com (R.I.); donatella.pastore@uniroma5.it (D.P.); david.dellamorte@uniroma2.it (D.D.-M.); 2 Section of Diabetes & Metabolic Disorders, UniCamillus, Saint Camillus International University of Health Sciences, 00131 Rome, Italy; 3 Department of Systems Medicine, University of Rome Tor Vergata, 00133 Rome, Italy; pacifici.francesca@gmail.com (F.P.); g.malatesta@outlook.it (G.M.); tesauro@med.uniroma2.it (M.T.); 4 Department of Clinical Sciences and Translational Medicine, University of Rome Tor Vergata, 00133 Rome, Italy; donadel@uniroma2.it; 5 Department of Neurology, Evelyn F. McKnight Brain Institute, University of Miami Miller School of Medicine, Miami, FL 33136, USA

**Keywords:** extra virgin olive oil, EVOO, polyphenols, oleocanthal, cancer, anticancer activity, neurodegeneration, neuroprotection, inflammation, oxidative stress

## Abstract

Dietary consumption of olive oil represents a key pillar of the Mediterranean diet, which has been shown to exert beneficial effects on human health, such as the prevention of chronic non-communicable diseases like cancers and neurodegenerative diseases, among others. These health benefits are partly mediated by the high-quality extra virgin olive oil (EVOO), which is produced mostly in Mediterranean countries and is directly made from olives, the fruit of the olive tree (*Olea europaea* L.). Preclinical evidence supports the existence of antioxidant and anti-inflammatory properties exerted by the polyphenol oleocanthal, which belongs to the EVOO minor polar compound subclass of secoiridoids (like oleuropein). This narrative review aims to describe the antioxidant and anti-inflammatory properties of oleocanthal, as well as the potential anticancer and neuroprotective actions of this polyphenol. Based on recent evidence, we also discuss the reasons underlying the need to include the concentrations of oleocanthal and other polyphenols in the EVOO’s nutrition facts label. Finally, we report our personal experience in the production of a certified organic EVOO with a “Protected Designation of Origin” (PDO), which was obtained from olives of three different cultivars (*Rotondella*, *Frantoio,* and *Leccino*) harvested in geographical areas located a short distance from one another (villages’ names: Gorga and Camella) within the Southern Italy “Cilento, Vallo di Diano and Alburni National Park” of the Campania Region (Province of Salerno, Italy).

## 1. Introduction: Extra Virgin Olive Oil (EVOO) Polyphenols

The consumption of olive oil represents a key pillar of the Mediterranean diet. A large body of evidence supports the positive effects of the Mediterranean diet on human health, spanning from the prevention of cardiometabolic disorders and cancers to neuroprotective properties [1,2,3,4]. In particular, a substantial fraction of such health benefits of the Mediterranean diet are mediated by the high-quality extra virgin olive oil (EVOO) [5,6], which is produced directly from olives, the fruit of the olive tree (*Olea europaea* L.), through mechanical extraction consisting of crushing and pressing the olives [5]. Indeed, olive oil consumption (particularly the extra virgin variety) has been associated with a reduced risk of cardiovascular disease and mortality in subjects at high cardiovascular risk in the PREDIMED study [7]. Therefore, EVOO is considered one of the highest-value food products and is mostly manufactured in Mediterranean countries. In the classic context of the Mediterranean diet, EVOO has always been one of the main sources of lipid intake, with a widespread presence in the traditional diet of all countries bordering the Mediterranean Basin. 

To date, an EVOO that meets specific requirements can be presented to the consumer with information on the label about its effects on human health. In the European Union (EU), there is an authorized health claim that “olive oil polyphenols contribute to the protection of blood lipids from oxidative stress” based on human studies documenting significantly reduced circulating levels of oxidized low-density lipoprotein (LDL) after virgin olive oil consumption [8]. This claim and other health claims are regulated by laws following the evaluations of regulatory agencies such as the European Food Safety Authority (EFSA) [9]. This claim may be used only for olive oil containing at least 5 mg of hydroxytyrosol and its derivatives (e.g., oleuropein complex and tyrosol) per 20 g of olive oil [8,9]. This legislation underscores the nutraceutical value attributed to EVOO by virtue of its high content of polyphenols, which are molecules that exert a series of beneficial effects on human health going beyond those traditionally attributed to oleic acid, a monounsaturated omega-9 fatty acid representing the main fatty acid of the olive oil.

To better understand the peculiar individual roles of each and every metabolite, researchers have attributed a leading role among these bioactive molecules to the oleocanthal in the group of secoiridoids—the same to which oleuropein belongs—due to its promising beneficial antioxidant and anticancer properties [10]. In an attempt to determine the degree of dose-dependent effects, attention has also shifted to the extraction methods of the EVOO since cultivation, harvesting and milling processes are critical factors that affect the resulting concentration of phenolic compounds in the ultimate product. As a matter of fact, new points of interest in this path are the molecular precursors in the fruit of the olive tree, the agronomic and transformation factors alongside the mechanisms of absorption, bioavailability and bioactivity of molecules such as oleocanthal, which make EVOO a food with different and multifaceted nutraceutical properties.

Therefore, in this narrative review, we aim to describe the antioxidant and anti-inflammatory properties of oleocanthal, which underlie its potential anticancer and neuroprotective actions. Based on recent evidence, we also discuss the reasons underlying the need to include the concentrations of oleocanthal and other polyphenols in the EVOO’s nutrition facts label. Moreover, we report our personal experience in the production of a certified organic EVOO with a “Protected Designation of Origin” (PDO), which was obtained from olives of three different cultivars in the Southern Italy “Cilento, Vallo di Diano and Alburni National Park” (Italian name: “Parco Nazionale del Cilento, Vallo di Diano e Alburni”) of the Campania Region (Province of Salerno, Italy). The PDO label is an EU quality certification that protects foods, agricultural products and wines made within specific geographical areas, under specific physical and biological conditions, and using well-defined production practices [11].

## 2. Classic EVOO Composition 

Functional compounds such as alkaloids or phenolic molecules are widely spread in plants, and the content profile of such molecules varies across different plant species. In the case of olive oil, the set of secondary metabolites is generally defined as that of the “minor compounds,” such as phenolic compounds and lipophilic compounds (like α-tocopherol or vitamin E), in order to distinguish them (constituting about 1–2% of the EVOO composition) from the more abundant molecules of the lipid fraction (also called “saponifiable fraction” or “glyceride fraction”) constituting about 98% of the EVOO composition. The saponifiable fraction is composed of fatty acids (mainly in the form of triglycerides) [12] such as oleic acid (monounsaturated omega-9 fatty acid accounting for about 55% and up to 83% of the total fatty acid content of the olive oil, depending on the reference cultivar and on the extraction techniques), linoleic acid (omega-6 polyunsaturated fatty acid accounting for 3–21% of the total fatty acid content of the olive oil), α-linolenic acid (omega-3 polyunsaturated fatty acid accounting for less than 1% of the total fatty acid content of the olive oil), and palmitic acid (long-chain saturated fatty acid accounting for 10–23% of the total fatty acid content of the olive oil) [13,14].

The definition of “minor polar compounds” [15] was established based on the relative hydrophilic properties of the molecules included in this class. Minor polar compounds are divided into different subclasses on the basis of their chemical structure. The most investigated subclasses are the subclass of phenolic secoiridoids—to which oleuropein and oleocanthal belong—and the subclass of phenylethanoids, which includes tyrosol and hydroxytyrosol. As previously mentioned, it is based on the content of hydroxytyrosol and its derivatives (e.g., oleuropein complex and tyrosol) that the EFSA allows the nutraceutical designation for an olive oil [9]. The other subclasses of phenolic acids (with trace active molecules such as gallic acid, protocatechuic acid, p-coumaric acid, and ferulic acid, among others), lignans (such as pinoresinol) and flavonoids (such as luteolin and apigenin) are also considered of importance [16,17]. Mention should be made of the numerous recent investigations conducted on the beneficial properties exerted by the latter three subclasses of compounds against different disorders, which, however, are not the exclusive trait of the phytochemical profile of EVOO but are instead found in a large variety of plant species suitable for human consumption. Table 1 lists the different subclasses of the main minor polar compounds found in EVOO. Among the main bioactive molecules belonging to the group of secoiridoids, there are oleuropein and oleocanthal, while the group of phenylethanoids includes tyrosol and hydroxytyrosol.

## 3. Oleocanthal Chemical Structure

**Figure 1:**
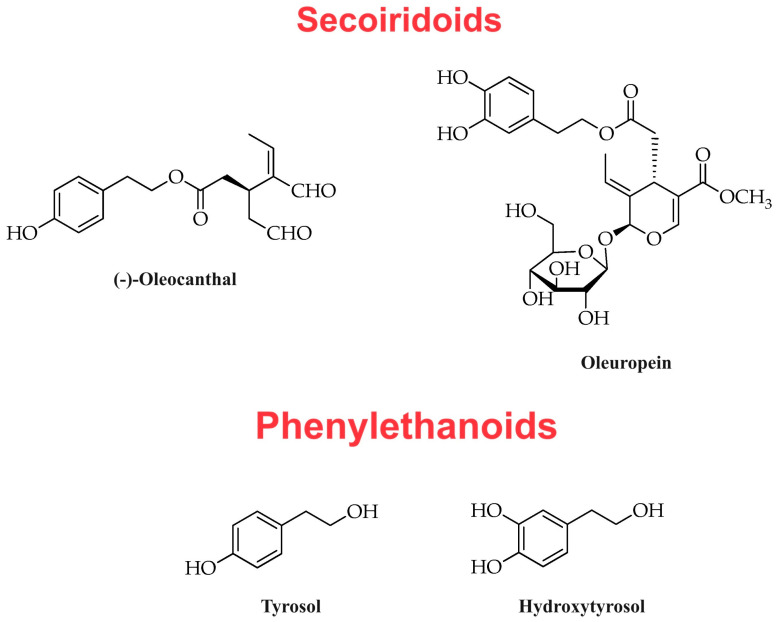
Chemical structure of the main polyphenols found in extra virgin olive oil (EVOO): secoiridoids and phenylethanoids.

**Figure 2:**
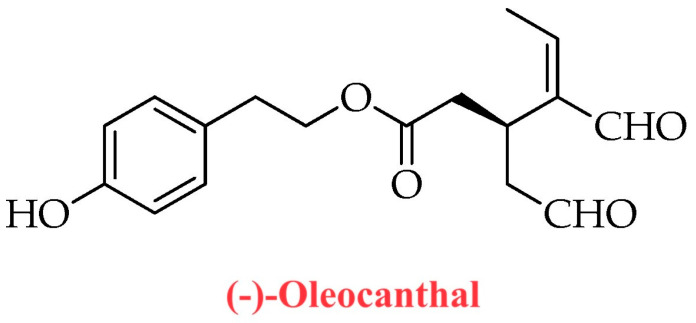
Chemical structure of oleocanthal.

The oleocanthal molecule, chemically known as the dialdehydic form of decarboxymethyl ligstroside aglycone (p-HPEA-EDA), was isolated and described in the scientific literature for the first time in the early 1990s as a phenolic compound [18]. At the molecular level, oleocanthal is a carboxylic ester, which is the 2-(p-hydroxyphenyl)ethyl ester of (3S)-4-formyl-3-(2-oxoethyl)hex-4-enoic acid [19]. Oleocanthal is not present (or is present at very low concentrations) in *Olea europaea* L. plants (leaves and fruits), but its production occurs as a result of multiple enzymatic reactions during EVOO production and processing (crushing and malaxation) [20,21]. Although oleocanthal belongs to the subgroup of secoiridoids, in the chemical structure of this naturally occurring phenolic secoiridoid (isolated from EVOO) it is possible to outline the hydroxylated phenethyl alcohol unit of tyrosol and hydroxytyrosol molecules, which belong to the subclass of phenylethanoids (Figure 1 and Figure 2). Oleocanthal was also identified as the main molecule responsible for the typical pungency that is generally associated with the tasting of EVOOs rich in polyphenols [22,23], which are cold-pressed extracts obtained (a few hours after olive harvest) solely through mechanical means from olives that are traditionally at the very beginning of their ripening phase (the so-called “first veraison”). An interesting aspect to be considered is that the spatial irritation produced by oleocanthal is specific to the oropharyngeal region, unlike irritants or pungent compounds that affect the perception of the entire oral cavity. Indeed, the term “oleocanthal” is composed of “*oleo*-”, which stands for olive, “-*canth*-”, which stands for stinging sensation, and “-*al*”, which stands for the presence of two aldehyde groups in the chemical structure of the molecule [23,24]. Thus, researchers suspected the existence of a specific sensory receptor for oleocanthal in the oropharyngeal region [25] and subsequently identified the Transient Receptor Potential Ankyrin 1 (TRPA1) non-selective cation channel as the oleocanthal sensory receptor [22]. In particular, oleocanthal and the non-steroidal anti-inflammatory drug ibuprofen have been identified as restricted throat irritants that selectively activate TRPA1 [22]. The marked interindividual variability of tissue sensitivity to oleocanthal within the oral cavity may be due to a high interindividual variability of TRPA1 receptor expression (and consequent oleocanthal-mediated TRPA1 activation) within the oropharyngeal region. Such observations began to validate oleocanthal as the molecule of choice for evaluating the quality of EVOOs with a high content of polyphenols. In this regard, the pungent sensation experienced upon EVOO ingestion suggests the presence of a high concentration of oleocanthal in the finished product rather than a high concentration of total polyphenols.

## 4. Antioxidant and Anti-Inflammatory Properties of Oleocanthal

Since the EVOO polyphenolic fraction is often tested for its recognized antioxidant activity as a whole, there is a paucity of studies investigating specifically the antioxidant capacity and redox potential of oleocanthal. However, preliminary preclinical evidence supports the antioxidant and anti-inflammatory properties of oleocanthal [24]. A study published in 2007 documented the total quantity of phenolic compounds in an Italian EVOO obtained from olives harvested in the production area of the province of Siracusa (Sicily, Italy). The content of total phenols—expressed as gallic acid equivalents—ranged from 14.80 to 121.20 mg/100 g (mean value: 53.72 mg/100 g) and was mainly attributable to deacetoxyligstroside aglycone, oleuropein aglycone, deacetoxyoleuropein aglycone, and ligstroside aglycone. Trolox equivalent antioxidant capacity (TEAC) and oxidative stability were both positively correlated with the phenolic content [26]. A recently published study conducted by Cuffaro et al. [27] investigated the antioxidant effects of the high-value polyphenol oleocanthal and its main metabolites (tyrosol and oleocanthalic acid) in terms of scavenging capacity towards biologically relevant reactive oxygen species (ROS). Remarkably, authors demonstrated that oleocanthal and tyrosol act as ROS scavengers, with a pronounced scavenging capacity towards superoxide anion radical (O2^●−^) and hypochlorous acid (HOCl) [27]. In addition, authors assessed the permeability of oleocanthal, tyrosol and oleocanthalic acid through the intestine in an intestinal co-culture model composed of Caco-2 and HT29-MTX cell lines, proving that such molecules exhibit a good intestinal permeation capacity (≥50% of intestinal permeation) [27].

**Figure 3:**
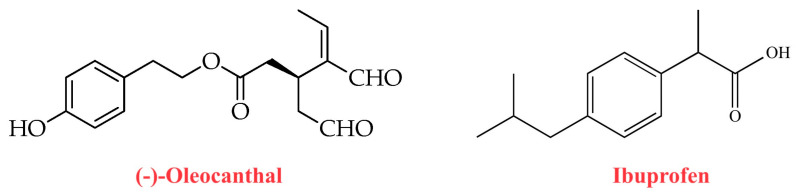
Comparison between the chemical structure of oleocanthal (on the **left**) and the chemical structure of the non-steroidal anti-inflammatory drug ibuprofen (on the **right**).

**Figure 4:**
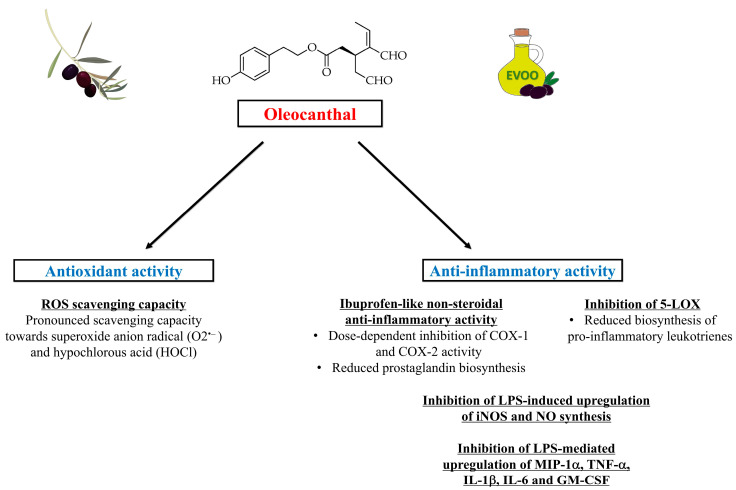
Molecular mechanisms underlying the antioxidant and anti-inflammatory actions of oleocanthal contained in extra virgin olive oil (EVOO). Abbreviations: 5-LOX, 5-lipoxygenase; COX-1, Cyclooxygenase-1; COX-2, Cyclooxygenase-2; EVOO, Extra virgin olive oil; GM-CSF, Granulocyte-macrophage colony-stimulating factor; IL-1β, Interleukin-1β; IL-6, Interleukin-6; iNOS, Inducible nitric oxide synthase; LPS, Lipopolysaccharide; MIP-1α, Macrophage inflammatory protein-1 alpha; NO, Nitric oxide; ROS, Reactive oxygen species; TNF-α, Tumor necrosis factor alpha.

Importantly, oleocanthal has also been shown to exert a natural non-steroidal anti-inflammatory activity. In this regard, Beauchamp et al. [23] documented that the strong stinging sensation in the throat induced by oleocanthal contained in newly pressed EVOO was similar to that caused by solutions of the non-steroidal anti-inflammatory drug ibuprofen. These findings indicated a shared pharmacological anti-inflammatory activity between the two molecules (oleocanthal and ibuprofen) [23]. Oleocanthal and ibuprofen also share similar substituents at para-position on the central phenyl group, which may result in a similar steric encumbrance (Figure 3). Ibuprofen is a non-selective inhibitor of the cyclooxygenase enzymes COX-1 and COX-2 involved in the prostaglandin-biosynthesis pathway [28]. Oleocanthal has been shown to act as a natural anti-inflammatory molecule with a potency markedly similar to that of ibuprofen, causing (like ibuprofen) a dose-dependent inhibition of COX-1 and COX-2 activity [23]. Oleocanthal has also been shown to inhibit 5-lipoxygenase (5-LOX) [29], the enzyme catalyzing the initial steps of the biosynthesis of pro-inflammatory leukotrienes, which are lipid mediators of inflammation derived from arachidonic acid [30]. Moreover, a study aimed to assess the anti-inflammatory activity of oleocanthal in murine macrophages found that this molecule can inhibit the lipopolysaccharide (LPS)-induced upregulation of inducible nitric oxide synthase (iNOS) and nitric oxide (NO) synthesis, as well as the LPS-mediated upregulation of inflammatory mediators such as macrophage inflammatory protein-1 alpha (MIP-1α), tumor necrosis factor alpha (TNF-α), interleukin-1β (IL-1β), interleukin-6 (IL-6) and granulocyte-macrophage colony-stimulating factor (GM-CSF) [31]. Figure 4 illustrates the molecular mechanisms underlying the antioxidant and anti-inflammatory actions of oleocanthal.

The hypothesis of antioxidant and anti-inflammatory actions exerted by oleocanthal has been tested in preclinical studies involving chronic degenerative disorders in which oxidative stress and inflammation play an important pathophysiological role, such as cancer and neurodegenerative disorders [32,33]. The first results of studies aimed at understanding the specific actions of oleocanthal—rather than those of the total polyphenolic fraction of EVOO—have shown a promising picture of efficacy towards the aforementioned conditions, as we will report later in the text. 

## 5. Anticancer Properties of Oleocanthal

Oleocanthal has been shown to exert promising anticancer actions on different cancer cell lines through a multitude of mechanisms, such as the modulation of signaling pathways like the apoptotic pathway, the HGF (Hepatocyte growth factor)/c-Met (Mesenchymal-epithelial transition factor) pathway, and the signal transducer and activator of transcription 3 (STAT3) pathway, among others [10]. The anti-inflammatory actions of oleocanthal may also partly account for its anticancer properties since chronic inflammation and COX-2 activity have been suggested to contribute to the so-called “inflammogenesis of cancer” by favoring carcinogenesis and tumor growth, angiogenesis, invasion and metastasis [34,35,36]. Furthermore, oleocanthal has been shown to exert synergistic effects with anticancer drugs [10]. Evidence for oleocanthal anticancer activity regards various cancer types associated with high mortality rates in humans. Herein, we summarize the emerging evidence for oleocanthal anticancer actions targeting melanoma, breast cancer, prostate cancer, pancreatic cancer, hepatocellular carcinoma, colorectal cancer, lung cancer, and hematologic malignancies such as multiple myeloma, acute promyelocytic leukemia, and chronic lymphocytic leukemia.

### 5.1. Melanoma

Oleocanthal has been shown to exert cytotoxic effects against human malignant melanoma cells [37]. Of note, Fogli et al. [37] documented that oleocanthal (at a concentration of 10 µM) exhibits a remarkable and selective in vitro antiproliferative activity against human melanoma cells (A375 and 501Mel cell lines), an effect that was accompanied by the inhibition of extracellular signal-regulated kinase 1/2 (ERK 1/2) and AKT (Protein kinase B) phosphorylation, and by the downregulation of the anti-apoptotic protein Bcl-2 (B-cell lymphoma 2) expression. Subsequently, an in vitro and in vivo study conducted by Gu et al. [38] demonstrated that oleocanthal (at concentrations up to 60 μM) suppressed proliferation, migration and invasion of A375 and A2058 melanoma cells, induced apoptosis of A375 and A2058 melanoma cells in a caspase-dependent manner, and inhibited metastasis of melanoma in a murine model of lung metastasis treated with 15 mg of oleocanthal/kg/day (administered intraperitoneally for 6 weeks). At a molecular level, oleocanthal suppressed the phosphorylation of the transcription factor STAT3, reduced STAT3 nuclear localization, and curbed STAT3 transcriptional activity, thereby downregulating STAT3-regulated gene products, including invasion-related proteins [matrix metalloproteinase (MMP)-2 and MMP-9], anti-apoptotic proteins (Bcl-xL and Mcl-1) and the angiogenic factor VEGF (Vascular endothelial growth factor), which are involved in invasion, apoptosis and angiogenesis of melanoma [38].

### 5.2. Breast Cancer, Prostate Cancer and Pancreatic Cancer

It has been documented that oleocanthal has marked antiproliferative effects against breast cancer cells. Akl et al. [39] conducted a study aimed at defining the intracellular mechanisms underlying the anticancer effects of oleocanthal in breast cancer. The authors found that oleocanthal treatment (at concentrations up to 25 µM) was able to inhibit the in vitro growth of human breast cancer cell lines MDA-MB-231, BT-474 and MCF-7. Additionally, oleocanthal caused a dose-dependent inhibition of HGF-induced cell migration, invasion and G1/S cell cycle progression in breast cancer cell lines. Notably, oleocanthal effects were driven by the inhibition of HGF-induced activation of c-Met and its downstream mitogenic signaling pathways. The aforementioned growth inhibitory effect was associated with the blockade of epithelial-mesenchymal transition (EMT) and with decreased cellular motility. Noteworthy, oleocanthal (administered intraperitoneally at a dose of 5 mg/kg/day; 3X/week) also suppressed tumor cell growth in an orthotopic model of breast cancer in athymic nude mice, without causing adverse effects on mice body weight or other clinical symptoms. These findings suggested that oleocanthal lacks potential systemic toxicity in athymic nude mice [39]. In keeping with these findings, Elnagar et al. [40] showed that oleocanthal inhibited the in vitro proliferation, migration and invasion of the epithelial human breast cancer cell lines MCF7 and MDA-MB-231, with a half-maximal inhibitory concentration (IC_50_) range of 10–20 µM. Similar effects have also been observed in prostate cancer cell lines PC-3 [40]. Khanfar et al. [41] documented that oleocanthal was able to inhibit the mTOR (mammalian target of rapamycin) signaling pathway in the breast adenocarcinoma cell line MCF-7 and in the human ductal breast epithelial tumor cell line T47D, with an IC_50_ value of 28.0 µM and 20.0 µM, respectively. Authors also found that oleocanthal (at a concentration of 10 µM) downregulated the expression of phosphorylated mTOR in the metastatic breast adenocarcinoma cell line MDA-MB-231 [41]. Moreover, LeGendre et al. [42] investigated the in vitro effects of oleocanthal on human cancer cell lines in culture [MDA-MB-231 (breast), PC3 (prostate), and BxPC3 (pancreatic) cancer cells], showing that oleocanthal treatment (at concentrations of 20 μM, in the absence of serum) induced a loss of cell adhesion within 30 min and resulted in 100% non-viability in all cancer cell lines after 24 h of treatment. Oleocanthal caused primary necrotic and apoptotic cell death by inducing lysosomal membrane permeabilization. Specifically, oleocanthal promoted lysosomal membrane permeabilization through inhibition of acid sphingomyelinase activity, which disrupts the interaction between proteins essential for lysosomal membrane stability. These findings support the role of oleocanthal as a potential lysosomotropic agent aimed to induce cancer-specific cell death. Importantly, oleocanthal treatment did not induce cell death in non-cancerous BJ human fibroblasts, 3Y1 rat fibroblasts and IMR90 human lung fibroblasts, but rather induced a reversible cell cycle arrest in these cells via suppression of Rb (retinoblastoma protein) phosphorylation, which is a mechanism thought to protect healthy cells against oleocanthal-mediated cell death [42].

In the context of breast cancer, oleocanthal has also been shown to exert synergistic effects with anticancer drugs. Siddique et al. [43] investigated the in vitro and in vivo synergistic antiproliferative effects of oleocanthal and lapatinib (a dual EGFR and HER2 tyrosine kinase inhibitor approved for treatment of advanced HER2-positive breast cancer) in BT-474 and SK-BR-3 human breast cancer cell lines and in an orthotopic xenograft tumor model of BT-474 cells in nude mice. Combination therapy with oleocanthal (at concentrations of 12–15 μM) and lapatinib (at doses of 30–60 nM) resulted in synergistic antiproliferative effects against the HER2 (Human epidermal growth factor receptor 2)-positive BT-474 and SK-BR-3 human breast cancer cell lines and significantly inhibited EGFR (Epidermal growth factor receptor), HER2 and c-Met receptor activation, as compared to oleocanthal monotherapy or lapatinib monotherapy. Combination oleocanthal and lapatinib therapy also significantly inhibited the invasion and migration of breast cancer cells by reducing the activation of focal adhesion kinase (FAK) and paxillin. Moreover, oleocanthal showed minimal effects on the viability of the non-tumorigenic mammary epithelial MCF-12A cells. In an orthotopic xenograft tumor model of BT-474 cells in nude mice, 60-day co-administration of 12.5 mg/kg lapatinib (oral, 5X/week) plus 10 mg/kg oleocanthal (intraperitoneal, 3X/week) resulted in the greatest tumor growth inhibition (suppression of more than 90% of BT-474 tumor cell growth), as compared to the vehicle and monotherapy controls. Western blot analysis of isolated mouse tumors collected at the end of the study revealed reduced total levels and activation of HER2, EGFR and c-Met in the combination-treated group. Importantly, the combination therapy lacked systemic toxicity, as documented by the absence of gross changes in the body weight of treated mice [43]. These results indicate that oleocanthal, as a c-Met inhibitor, could represent a safe and effective adjuvant agent in anticancer combination therapies aimed to selectively sensitize HER2-overexpressing breast cancer cells (without affecting the proliferation of non-cancerous cells), enhance the activity of HER2-targeted therapies (like lapatinib) and consequently reduce the required doses and toxicity of such therapies, as well as the possible emergence of HER2-targeted therapy resistance. Moreover, the same research group documented that oral oleocanthal treatment (at a dose of 10 mg/kg/day) for 40 days was effective in preventing breast cancer locoregional recurrence in a nude mouse xenograft model generated through orthotopic inoculation of BT-474 breast cancer cells (which represent the luminal B cancer subtype expressing hormone receptors and HER2) [44]. Authors also reported the inhibition of tumor recurrence by oleocanthal after the completion of neoadjuvant treatment with lapatinib (50 mg/kg; oral, 5X/week, for 19 days). There was no significant difference in the mean animal body weight between the vehicle-control and oleocanthal-treated groups throughout the study duration [44].

Siddique et al. [45] also demonstrated that a daily oral oleocanthal dose of 10 mg/kg for 11 days effectively suppressed the progression of the metastatic castration-resistant prostate cancer (mCRPC) CWR-R1ca cells engrafted into orthotopic athymic nude mice. Authors did not observe gross adverse and/or behavioral responses in male athymic nude mice treated with oleocanthal, and the mice’s mean body weight was not significantly different over the experiment course in placebo control- and oleocanthal-treated groups. Mice underwent primary tumor surgical excisions and continued to receive daily oral oleocanthal treatment for an additional 30 days. Daily oral oleocanthal administration for 30 days suppressed tumor locoregional and distant recurrences after primary tumor surgical excision. Western blot analysis of collected primary tumor lysates confirmed the reduction of the expression level of the histone methyltransferase SMYD2 (SET and MYND domain-containing protein 2) in oleocanthal-treated primary tumors, as compared to that observed in the placebo control-treated group. Moreover, a significant 15.9% reduction in the serum levels of the prostate cancer recurrence marker PSA (Prostate-specific antigen) was observed in the oleocanthal-treated mice, as compared to the placebo control-treated group. Thus, this study suggested SMYD2 as a novel molecular target in mCRPC and oleocanthal as a specific SMYD2 inhibitor [45].

### 5.3. Hepatocellular Carcinoma (HCC) and Colorectal Cancer (CRC)

Some studies showed promising antiproliferative effects exerted by oleocanthal against hepatocellular carcinoma (HCC) and colorectal cancer (CRC). Pei et al. [46] showed that oleocanthal (administered at increasing doses up to 80 μM for 24–72 h) inhibited proliferation, cell cycle progression, migration and invasion, and induced apoptosis in HCC cells in vitro. The same oleocanthal doses had no effect on the viability of normal human liver cells (LO2 cells), suggesting that this molecule specifically inhibits HCC progression. Oleocanthal also suppressed tumor growth in an in vivo orthotopic HCC murine model and counteracted HCC metastasis in an in vivo lung metastasis murine model. In the in vivo experiments, mice were treated with oleocanthal administered intraperitoneally (at a dose of 5 mg/kg/day or 10 mg/kg/day) for 5 weeks. Mechanistically, oleocanthal inhibited EMT by downregulating the expression of the transcription factor Twist (a direct target of STAT3) and by decreasing STAT3 nuclear translocation and DNA binding activity, ultimately downregulating the cell cycle protein cyclin D1, the anti-apoptotic proteins survivin and Bcl-2, as well as the invasion-related protein MMP-2 [46]. Cusimano et al. [47] demonstrated that oleocanthal, as compared to classical commercially available COX inhibitors (ibuprofen, nimesulide and indomethacin), was more effective in inducing inhibition of in vitro cell growth in a series of human HCC (HepG2, Huh7, Hep3B and PLC/PRF/5) and CRC (HT29 and SW480) cell lines. Oleocanthal treatment showed the strongest inhibition of cell viability in Hep3B cells with an IC_50_ value of 26.6 µM, followed by an IC_50_ value of 41.9 µM in HepG2 cells. Authors also documented that oleocanthal inhibited colony formation, induced apoptosis (confirmed by PARP [Poly (ADP-ribose) polymerase] cleavage, activation of caspases 3/7 and chromatin condensation), induced the expression of γH2AX histone (a well-known molecular marker of DNA damage), and led to mitochondrial depolarization [47]. Importantly, oleocanthal did not show toxicity in primary normal human hepatocytes even when administered at high doses (100 µM) for 72 h, indicating that this molecule exerts cytotoxic effects on liver cancer cells but has no effect on the cell viability of normal hepatocytes [47]. Finally, Khanal et al. [48] demonstrated that the dialdehydic form of decarboxymethyl ligstroside aglycone (p-HPEA-EDA)—administered at doses up to 10 μg/mL—suppressed COX-2 and tumorigenicity by activation of adenosine monophosphate-activated protein kinase (AMPK) in a human colorectal adenocarcinoma cell line (HT-29 cell line). These findings suggest that p-HPEA-EDA-mediated COX-2 inhibition and targeted activation of AMPK may contribute to the chemotherapeutic potential of EVOO against CRC.

### 5.4. Lung Cancer

Siddique et al. [49] conducted in vitro and in vivo experiments to investigate the oleocanthal’s ability to suppress lung cancer progression, migration and metastasis. Authors demonstrated that oleocanthal (at concentrations ranging between 10 and 30 µM) inhibited the HGF-mediated growth and migration in lung adenocarcinoma cells A549 and NCI-H322M through dual targeting of c-Met and COX-1/COX-2, reducing the total and activated c-Met levels and inhibiting COX-1/COX-2 activity (with a higher preference towards COX-2). On the other hand, oleocanthal treatment at a dose of 40 µM had little effect on the viability of immortalized non-tumorigenic human microvascular endothelial cells (HMVEC) versus vehicle-treated control groups. Moreover, 8-week oral treatment with oleocanthal (at a dose of 10 mg/kg/day) significantly suppressed the human non-small cell lung cancer cell (A549-Luc cells) progression and metastasis to the brain, heart, kidneys and spleen in a lung cancer nude mouse tail vein injection model. Notably, authors monitored the mice’s body weight over the course of the study without detecting any change between oleocanthal and vehicle control treatments. Human microarray analysis revealed that oleocanthal-treated A549-Luc lung cancer tissues had a distinct gene signature that confirmed the dual targeting of c-Met and COX-2, thus supporting the potential role of oleocanthal as a valid adjuvant agent for the prevention and treatment of lung cancer [49].

### 5.5. Multiple Myeloma, Acute Promyelocytic Leukemia and Chronic Lymphocytic Leukemia 

An in vitro study conducted on the human multiple myeloma cell line ARH-77 by Scotece et al. [50] showed that oleocanthal (at doses ranging between 10 and 50 μM) exerts an antiproliferative activity by promoting apoptosis and by downregulating ERK1/2 and AKT signal transduction pathways in a dose-dependent manner. In the same study, oleocanthal (used at concentrations ranging between 10 and 50 μM) also caused a dose-dependent inhibition of MIP-1α expression in ARH-77 cells [50]. This finding is highly relevant since MIP-1α seems to be involved in the development of osteolytic lesions related to multiple myeloma [51].

Fabiani et al. [52] examined the in vitro effect of a complex phenol extract obtained from virgin olive oil on proliferation, cell cycle distribution profile, apoptosis and differentiation of the human promyelocytic HL60 leukemia cells. In addition, authors quantified the contribution to the response obtained with the whole phenol extract that was attributable to the single purified compounds 3,4-DHPEA-EDA and p-HPEA-EDA. Interestingly, p-HPEA-EDA was more potent than 3,4-DHPEA-EDA in suppressing cell growth, as the concentrations at which the aforementioned compounds caused 50% inhibition of cell proliferation were 7–8 μmol/L and 30–35 μmol/L, respectively. Similarly, p-HPEA-EDA was more potent than 3,4-DHPEA-EDA in causing apoptosis of HL60 cells 24 h after treatment, inducing apoptosis in 90% of the cells at a concentration of 60 μmol/L, while a concentration of 256 μmol/L of 3,4-DHPEA-EDA was needed to observe the same effect. At the highest concentrations, both p-HPEA-EDA and 3,4-DHPEA-EDA were cytotoxic, as documented by the increased percentage of necrotic cells [52]. 

With regard to clinical evidence, a pilot study conducted by Rojas Gil et al. [53] on 22 patients with early-stage chronic lymphocytic leukemia (CLL)—who did not meet the criteria for starting chemotherapy—tested the tolerability and the anticancer potential of a dietary intervention consisting in the daily consumption of 40 mL of an oleocanthal- and oleacein-rich EVOO (containing 416 mg/kg of oleocanthal and 284 mg/kg of oleacein) for 6 months. During the dietary intervention, authors observed a statistically significant decrease in the white blood cell (WBC) and lymphocyte count, as compared to the values observed 3 months before the intervention and 6 months after the beginning of the intervention. At 6 months from the beginning of the dietary intervention, authors also observed a significant increase in the serum expression levels of the apoptotic markers ccK18 and Apo1-Fas, a significant decrease in the serum expression levels of the anti-apoptotic protein survivin, a significant decrease in the peripheral blood mononuclear cells (PBMC) protein expression levels of survivin and cyclin D (a cell proliferation marker), as well as a significant increase in the PBMC protein expression levels of p21 (a negative regulator of the cell cycle). A significant increase in the percentage (40%) of apoptotic cells was also observed in PBMCs isolated from CLL patients at 3 and 6 months after the initiation of dietary intervention. Interestingly, authors also documented that patients who initially had higher WBC count values and higher protein expression levels of ccK18 and survivin exhibited a greater reduction of WBC count values, a greater increase of ccK18 levels, and a greater decrease of survivin levels after dietary intervention, as compared to those with lower initial WBC count values and lower initial protein expression levels of ccK18 and survivin, respectively. A significant negative correlation was also observed between the WBC count at the end of the dietary intervention, the initial ccK18 values, and the fluctuation in the protein expression of ccK18. These findings indicate the possible existence of circulating biomarkers able to predict the anticancer efficacy of oleocanthal in patients with CLL. Importantly, the consumption of the oleocanthal- and oleacein-rich EVOO was well tolerated and was not associated with side effects. Thus, this pilot study suggested that the daily consumption of 40 mL of an oleocanthal- and oleacein-rich EVOO (providing approximately 15 mg of oleocanthal and 10 mg of oleacein) may represent a safe adjuvant dietary intervention aimed to promote cancer cell apoptosis in patients with early-stage CLL [53].

**Figure 5:**
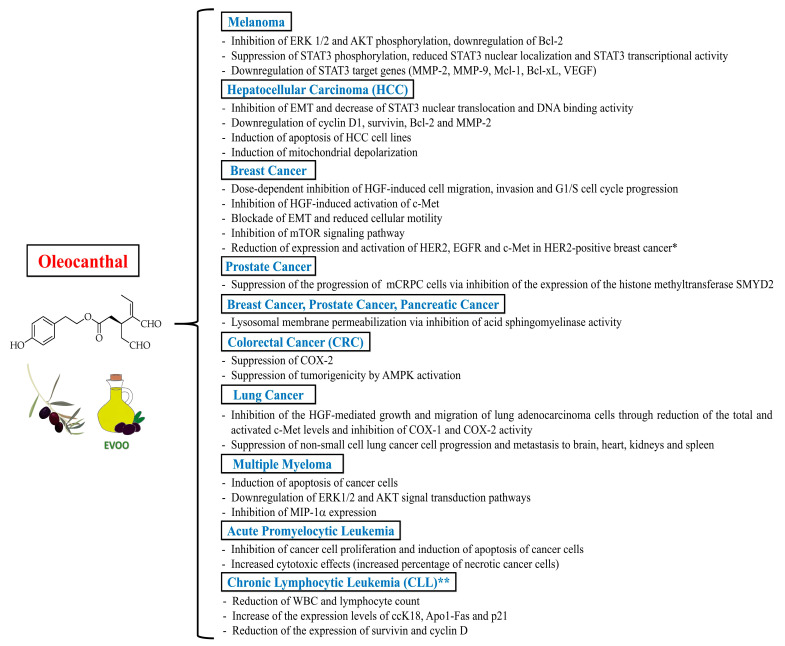
Anticancer actions exerted by oleocanthal (contained in extra virgin olive oil) against different cancer types. These actions have mostly been inferred from preclinical studies conducted in vitro and in vivo. Abbreviations: AKT, Protein kinase B; AMPK, Adenosine monophosphate-activated protein kinase; Bcl-2, B-cell lymphoma 2; Bcl-xL, B-cell lymphoma-extra large; c-Met, Mesenchymal-epithelial transition factor; CLL, Chronic lymphocytic leukemia; COX-1, Cyclooxygenase-1; COX-2, Cyclooxygenase-2; EGFR, Epidermal growth factor receptor; EMT, Epithelial-mesenchymal transition; ERK 1/2, Extracellular signal-regulated kinase 1/2; EVOO, Extra virgin olive oil; HCC, Hepatocellular carcinoma; HER2, Human epidermal growth factor receptor 2; HGF, Hepatocyte growth factor; Mcl-1, Myeloid cell leukemia-1 protein; mCRPC, Metastatic castration-resistant prostate cancer; MIP-1α, Macrophage inflammatory protein-1 alpha; MMP-2, Matrix metalloproteinase-2; MMP-9, Matrix metalloproteinase-9; mTOR, Mammalian target of rapamycin; SMYD2, SET and MYND domain-containing protein 2; STAT3, Signal transducer and activator of transcription 3; VEGF, Vascular endothelial growth factor; WBC, White blood cell. * Synergistic effects of oleocanthal and lapatinib (a dual EGFR and HER2 tyrosine kinase inhibitor). ** Anticancer actions inferred from a pilot clinical study conducted in patients with early-stage CLL.

Taken together, the abovementioned studies mostly provide preclinical evidence of the chemotherapeutic potential of oleocanthal in different cancer types. Remarkably, such studies documented that oleocanthal exhibits selective antiproliferative effects on different cancerous cells without substantially affecting the proliferation of normal cells and without causing adverse effects in vivo. The selective oleocanthal antiproliferative effects on cancerous cells may partly rely on the previously reported oleocanthal ability to inhibit the heat shock protein 90 (Hsp90) [54], a highly conserved molecular chaperone fulfilling a housekeeping function in contributing to the folding, maintenance of structural integrity and homeostatic regulation of a number of proteins [55], including some proteins required for tumor growth [56]. These findings are highly promising since there is a great need for novel targeted anticancer therapies causing selective cytotoxic effects on cancerous cells without influencing the cell cycle of non-cancerous cells. Nonetheless, future animal and human studies are needed to ascertain whether (and at which doses) oleocanthal has anticancer properties in vivo. Figure 5 illustrates the anticancer actions (mostly inferred from preclinical studies) exerted by oleocanthal against different cancer types.

## 6. Neuroprotective Properties of Oleocanthal

Neurodegenerative disorders represent a heterogeneous group of conditions characterized by the progressive dysfunction of selected populations of neurons [57]. Neuronal loss is accompanied by extracellular and intracellular accumulation of misfolded proteins (the hallmarks of different neurodegenerative proteinopathies such as tauopathies), which ultimately leads to programmed cell death [57]. Alzheimer’s disease is one of the most common neurodegenerative disorders [58] and is pathologically defined by the accumulation of fibrillar amyloid beta (Aβ) peptide in extracellular amyloid plaques and by the abnormal deposition of tau filaments in the form of intracellular neurofibrillary tangles [59]. Tau is a microtubule-associated protein that is abundant in the axons of neurons, where it regulates the microtubule assembly and the maintenance of microtubule structural stability [60]. In Alzheimer’s disease and related tauopathies, tau forms insoluble filaments that accumulate as neurofibrillary tangles (comprised of abnormal hyperphosphorylated tau aggregates) and contribute to the pathophysiology of such diseases [61]. Microglia and astrocytes also play an important role in the pathophysiology of Alzheimer’s disease, as these cells become activated around the Aβ plaques and neurofibrillary tangles in the brain and drive neuroinflammatory responses that, in turn, exacerbate the neurodegeneration and further increase the Aβ and tau overproduction, thus triggering a vicious cycle [62,63].

A growing body of preclinical evidence suggests that polyphenols (such as oleocanthal, hydroxytyrosol, tyrosol and resveratrol, among others) and their derivatives (e.g., tyrosol phosphodiester derivatives containing a catechol moiety) exert neuroprotective properties by counteracting the growth and neurotoxicity of Aβ plaques [64,65,66,67,68,69,70,71,72,73]. Therefore, these molecules may contribute to preventing cognitive decline related to neurodegenerative disorders such as Alzheimer’s disease.

With regard to oleocanthal, Li et al. [74] demonstrated that this molecule (at concentrations ranging between 1 μM and 100 μM) prevents the fibrillization of tau by locking tau into a naturally unfolded state. Since oleocanthal contains two aldehyde groups, authors assumed that tau protein may be covalently modified, specifically at the lysine residues. Indeed, authors revealed that oleocanthal forms an adduct with the lysine residues through initial Schiff base formation and subsequently inhibits tau fibrillization [74], which is one of the main causes of neurodegeneration in Alzheimer’s disease and related tauopathies [61]. Afterwards, Monti et al. [75] conducted a mass spectrometric investigation of the oleocanthal reactive profile with tau protein fibrillogenic fragment K18 and propylamine, documenting that K18 was prone to be covalently modified by oleocanthal via Schiff base formation between the oleocanthal aldehyde carbonyls and the ε-amino group of lysine residues. The same research group subsequently showed that oleocanthal interacts with tau-441 isoform, causing stable conformational changes in the protein secondary structure and counteracting tau aggregation [76].

Additional evidence supports a neuroprotective role of oleocanthal in Alzheimer’s disease based on its ability to enhance Aβ clearance from the brain. Abuznait et al. [64] showed that C57BL/6 wild-type mice treated with oleocanthal (administered intraperitoneally at a dose of 10 mg/kg twice daily for 2 weeks) exhibited an enhanced ^125^I-Aβ_40_ clearance from the brain and a significantly higher brain efflux index as compared to control mice. Interestingly, authors also demonstrated that oleocanthal significantly upregulated the Aβ degrading enzyme insulin-degrading enzyme (IDE) in C57BL/6 mice brain microvessels. Moreover, oleocanthal treatment (at increasing doses within the range of 0.5–50 μM for 72 h) increased P-glycoprotein (P-gp) and Low-density lipoprotein receptor-related protein 1 (LRP1) expression and activity in cultured mouse brain endothelial cells [64]. It is worth reminding that P-gp and LRP1 have been reported as major Aβ transport proteins mediating the Aβ removal from the brain across the blood-brain barrier [77,78]. Qosa et al. [65] investigated the effect of oleocanthal in TgSwDI mice, an animal model of Alzheimer’s disease. Mice treated with oleocanthal for 4 weeks (at a dose of 5 mg/kg/day, administered intraperitoneally) showed significantly decreased amyloid load in the hippocampal parenchyma and microvessels, as well as enhanced cerebral clearance of Aβ across the blood-brain barrier. Furthermore, oleocanthal enhanced the apolipoprotein E (ApoE)-dependent clearance pathway of Aβ via the activation of PPARγ (Peroxisome proliferator-activated receptor gamma) expression in mouse brain tissue. Authors also confirmed that oleocanthal treatment increased the expression of P-gp and LRP1 at the blood-brain barrier [65]. Batarseh et al. [79] tested the oleocanthal ability to alter the toxic effect of Aβ on cultures of human neurons and astrocytes. Authors demonstrated that oleocanthal (at a dose of 5 μM) prevented Aβ oligomers (Aβo)-induced neuronal downregulation of synaptic proteins SNAP-25 (Synaptosomal-associated protein of 25 kDa) and PSD-95 (Postsynaptic density protein 95), and mitigated Aβo-induced inflammation and downregulation of glutamate transporter-1 (GLT1) and glucose transporter-1 (GLUT1) in astrocytes [79]. Finally, a study conducted by Abdallah et al. [80] in homozygous 5xFAD mice (a preclinical model of Alzheimer’s disease characterized by an accelerated formation of amyloid plaques) demonstrated that a 3-month oleocanthal-enriched diet (with oleocanthal added at a dose of 0.5 mg/kg/day) decreased the brain Aβ levels and the neuroinflammation by suppressing the nuclear factor-κB (NF-κB) pathway and reducing the activation of the NLRP3 inflammasome. Moreover, oleocanthal suppressed the receptor for advanced glycation end products/high mobility group box 1 (RAGE/HMGB1) pathway [80], which is also involved in the activation of the NF-κB pathway and related neuroinflammation in Alzheimer’s disease [81].

The neuroprotective potential of oleocanthal may also rely on its antioxidant properties. Indeed, Giusti et al. [82] showed that oleocanthal treatment (at a concentration of 10 μM) counteracted oxidative stress in neuron-like SH-SY5Y cells exposed to hydrogen peroxide (H_2_O_2_) by increasing cell viability, reducing ROS production and increasing intracellular levels of reduced glutathione (GSH) [82]. Notably, the brain’s vulnerability to oxidative stress is an important contributor to the pathophysiology of Alzheimer’s disease. Indeed, Aβ accumulation induces oxidative stress, and oxidative stress increases the Aβ deposition [83].

It is also interesting to note that the anti-amyloidogenic properties of oleocanthal are similar to some of those exerted by resveratrol (3,5,4′-trihydroxy-trans-stilbene), a naturally occurring polyphenolic phytoalexin mainly found in grape skin, raspberries, mulberries and wine [84]. Indeed, in vitro and in vivo (animal) studies documented that resveratrol inhibits Aβ-mediated microglial activation, increases total brain LRP1 levels, decreases the levels of insoluble Aβ1-42 in the hippocampus, and attenuates Aβ-induced cytotoxicity, apoptosis and intracellular reactive oxygen intermediate (ROI) accumulation [71,85,86,87,88,89]. Interestingly, resveratrol has also been shown to interfere with the aggregation of human islet amyloid polypeptide (hIAPP) [72], a highly amyloidogenic polypeptide forming intracellular aggregates and amyloid deposits within the pancreatic islets that are associated with pancreatic beta-cell death [90]. Evidence suggests that IAPP may contribute to the pathophysiology of diabetes mellitus-related cognitive dysfunction and dementia by impairing the blood-brain barrier and interacting with Aβ peptide and with tau within the brain (cross-amyloid interactions) [90].

**Figure 6:**
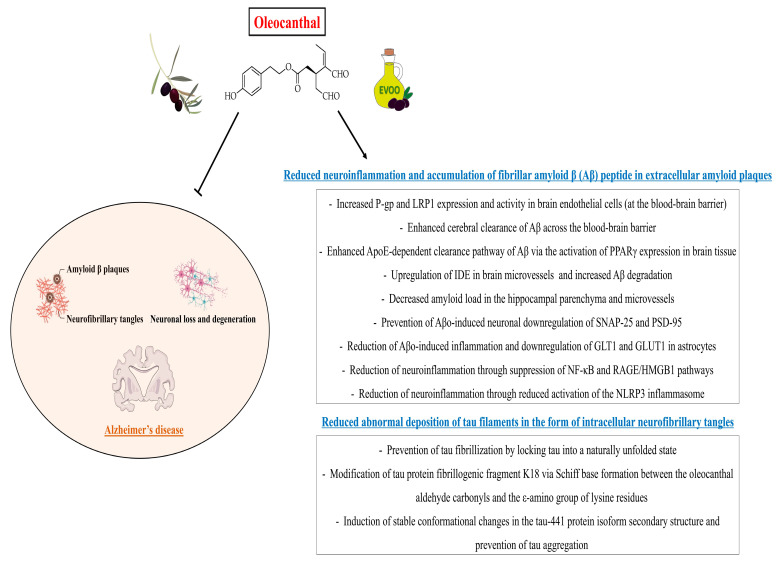
Potential neuroprotective actions exerted by oleocanthal (contained in extra virgin olive oil) that may be particularly beneficial for prevention and treatment of Alzheimer’s disease. The neuroprotective actions of oleocanthal have mainly been inferred from preclinical studies conducted in vitro and in vivo, where this molecule has been shown to exert protective properties against the pathological hallmarks of Alzheimer’s disease, such as accumulation of fibrillar amyloid beta (Aβ) peptide in extracellular amyloid plaques and abnormal deposition of tau filaments in the form of intracellular neurofibrillary tangles. Abbreviations: ApoE, Apolipoprotein E; Aβ, Amyloid beta peptide; Aβo, Aβ oligomers; EVOO, Extra virgin olive oil; GLT1, Glutamate transporter-1; GLUT1, Glucose transporter-1; IDE, Insulin-degrading enzyme; LRP1, Low-density lipoprotein receptor-related protein 1; NF-κB, Nuclear factor-κB; P-gp, P-glycoprotein; PPARγ, Peroxisome proliferator-activated receptor gamma; PSD-95, Postsynaptic density protein 95; RAGE/HMGB1, Receptor for advanced glycation end products/high mobility group box 1; SNAP-25, Synaptosomal-associated protein of 25 kDa.

Altogether, these findings suggest that dietary consumption of oleocanthal-rich EVOO may theoretically represent a promising nutritional tool for the prevention of neurodegenerative disorders such as Alzheimer’s disease. However, future animal and human studies are required to establish the potential of oleocanthal for the prevention and treatment of neurodegenerative disorders. Figure 6 illustrates the potential oleocanthal neuroprotective actions that may be particularly beneficial for the prevention and treatment of Alzheimer’s disease.

## 7. Oleocanthal as a Nutraceutical

Based on the aforementioned studies, it appears plausible that the consumption of oleocanthal-rich EVOO on a regular basis—even in dietary protocols outside of a traditional Mediterranean dietary regimen—may contribute to counteracting the onset and progression of several chronic non-communicable diseases, such as various cancer types and neurodegenerative disorders. Since the concentrations of different polyphenols (including oleocanthal) in olive fruits are variable based on distinct factors (cultivars, oil extraction methods, climatic conditions, etc.), several polyphenol supplements have been developed through novel technologies (e.g., encapsulation and nano-delivery systems) to enhance their oral bioavailability and to ensure an adequate daily intake of the aforementioned compounds [91]. Yet, even if the total polyphenol concentration in olive oil can be made more abundant with appropriate agricultural and extraction techniques, it cannot be excluded that the greater biological activity in this family is exerted by one or more specific isolable compounds. It was precisely on the basis of these premises that, in the first decade of the 2000s, the EFSA was asked to express an opinion on the nutritional properties of EVOO and its benefits for human health. In this regard, the European Parliament approved the Regulation No. 1924/2006, which governs the use of health claims on food products based on EFSA reports [92].

Nutraceutical EVOO polyphenols (increasingly widespread, especially on the European market) are generally purified starting from water waste resulting from milling procedures. In this regard, it is worth reminding that a substantial quantity of polyphenols is found in the olive vegetation water (OVW) [93], which constitutes a by-product of olive processing whose final objective is to remove the maximum amount of water from the fruit in order to increase the oil yield. Interestingly, hydrolyzed OVW has been shown to exert anti-inflammatory actions in mice [94]. The olive vegetation waters (OVWs) produced in the oil mill are made up of olive washing water, process water and the aqueous fraction of the drupe juices. Even though they have long been considered a waste product to be disposed of as a special residue, OVWs have now become part of the olive tree cycle as a valid resource in all respects. They are, in fact, rich in organic matter and contribute to combating the desertification of the soil. OVW spillage is appropriately regulated since the most significant limit to the spreading of OVWs on soils is primarily due to the OVW’s acidic character (with a pH value around 5). Moreover, the high level of total polyphenols contained in OVWs can slow down the transformation and biodegradation processes due to the polyphenols’ antimicrobial properties [93].

If we intend to explore the potential of polyphenols primarily in the food industry, we must return to the importance of all agricultural and transformative processes that are studied with the precise aim of producing EVOOs with a high concentration of bioactive compounds. In recent decades, such processes have seen an ever-increasing impetus in the wake of a renewed awareness towards healthy and nutrient-dense foods. Nowadays, the indications on the label for high-quality EVOO have become more familiar, as they inform consumers about the exclusive employment of mechanical processes or about the oil having been obtained solely from cold-pressed olives, just to give a few examples. Yet, the practice of cold olive oil extraction is certainly an important contributor to the total polyphenol yield. Cold-pressed or cold-extracted EVOO claims apply only to EVOOs obtained at a temperature lower than 27 °C [95,96]. Polyphenols are regarded as heat-labile compounds, and thermal degradation represents the most common explanation for the degradation of such compounds [97].

It is worth noting that the concentration of phenolic compounds in EVOOs usually ranges between 100 and 300 mg/kg, with oleocanthal representing approximately 10% of the total EVOO phenolic compounds [18,98]. The aforementioned total phenolic content (TPC) of EVOO amounts to 10–30 mg/100 g oil, which is substantially higher than that reported in other edible vegetable oils such as groundnut oil (3.09 mg/100 g oil), coconut oil (1.8 mg/100 g oil), rice bran oil (0.89 mg/100 g oil), mustard oil (0.56 mg/100 g oil), sunflower oil (0.49 mg/100 g oil) and sesame oil (0.33 mg/100 g oil) [99]. Thus, the high TPC makes EVOO an ideal edible vegetable oil whose dietary consumption can be exploited to successfully achieve the human health benefits of polyphenols (including oleocanthal). 

In the multicenter, randomized controlled trial PREDIMED, including 7216 men and women at high cardiovascular risk (aged 55 to 80 years), study participants were randomized to one of three different interventions: Mediterranean diets supplemented with nuts or EVOO, or a control low-fat diet [7]. The mean intake of total olive oil was 56.9 g/day in participants allocated to the highest tertile, compared to 21.4 g/day in participants allocated to the lowest tertile [7]. Each increase of 10 g/day in EVOO intake was associated with a 10% decrease in the risk of cardiovascular events [7]. It has been postulated that the reasonable daily intake of 25–50 mL olive oil provides no more than 0.9 mg of oleocanthal (corresponding to about 5–10 teaspoons) [24,98]. Consequently, the concentrations of oleocanthal found within an usual amount of EVOO consumed on a daily basis (25–50 mL) are likely insufficient for the occurrence of the antioxidant and anti-inflammatory oleocanthal effects in vivo [98].

Nevertheless, in recent years, functional olive oils with a high concentration of selected polyphenols have become increasingly available on the market. These are polyphenol-rich EVOOs that have benefited from the correct application of the best practices in agriculture that go hand in hand with better optimized machinery for delicate processing in complete automation. Yet, even before the milling of olives, the fine regulation of the production yield is influenced by the organoleptic profile of the many varieties of this botanical species, by the orographic characteristics of the land where *Olea europaea* L. plants grow, and by the climatic conditions of the year. Moreover, the ability to activate biochemical defenses against the frequent attacks from olive tree pests is also an important factor for obtaining an optimal EVOO polyphenolic profile. Therefore, we decided to add to our discussion the recent results regarding the extraction processes and the variability of the active metabolites between different cultivars. Later in the text, we will also provide the example of a typical quality certification of an Italian nutraceutical EVOO, with laboratory results regarding the concentrations of distinct polyphenols. This type of analysis is generally considered routinary for the issuance of the PDO (“Protected Designation of Origin”) quality mark certification (Italian acronym: DOP, “Denominazione di Origine Protetta”), which is assigned on the basis of specific laboratory tests coupled with the assessment of the olive oil organoleptic characteristics (sensory analysis score) conducted by a panel of experts. This is the human component that establishes the assignment of the EVOO designation. At the end of the evaluation, scores are assigned for possible defects and for fruity, bitter and spicy sensations evoked by EVOO ingestion, which are precious tools in the EVOO’s quality appraisal. Traditionally, the sensation of spiciness has always been associated with a high polyphenol content during panel evaluations. Unusual oral pungency from EVOO has been attributed to restricted spatial expression of TRPA1, which mediates the tissue sensitivity to oleocanthal within the oral cavity [22], as we previously mentioned. If further investigations confirm that the oral pungency sensation evoked upon EVOO ingestion is mainly due to oleocanthal, new evaluation scores could also be modified accordingly to the point where an oil with a high median spiciness would be highly suitable for being defined as an oleocanthal-rich EVOO prior to its thorough chemical analysis.

### 7.1. Oleocanthal-Rich EVOO

In order to understand the factors determining the final yield of olive oil, we previously mentioned the meteorological conditions and the characteristics of the territory on which the different cultivars thrive. Some agricultural research works have highlighted how the various stages of growth and oil content for different varieties of olives are strongly influenced by climate and by the ability of the botanical species in question to adapt to it. In a study conducted from 2003 to 2009 in the Southern Italy Campania Region, which is characterized by favorable climatic conditions and by a robust varietal heritage, 20 cultivars belonging to Campania’s olive germplasm were thoroughly examined in terms of vegetative and production aspects and oil quality characteristics [100]. Authors examined various cultivar aspects such as entry into production, vigor, ripening and drupe oil content. Agronomic results showed an early entry into production for cultivars such as *Biancolilla* and *Carpellese* (from the Province of Salerno), as compared to others located both in distant and nearby territories, such as *Ortice* (from the Province of Benevento) and *Rotondella* (from the Province of Salerno). *Asprinia* (from the Province of Caserta), *Pisciottana* and *Carpellese* (from the Province of Salerno) were the most vigorous cultivars. Thus, researchers emphasized the strong and diverse typicality of olive tree varieties even within a single Italian region [100].

In parallel with the final oil yield, the EVOO’s TPC is strongly influenced by various factors such as climate, soil characteristics, extraction techniques, quantification methodologies, malaxation conditions and harvest period [21,101,102]. This aspect has relevant implications, since it is possible to increase the content of specific polyphenols (such as oleocanthal) in the final olive extract thanks to specific methods of harvesting and processing the fruit of the olive tree. A recent study by Diamantakos et al. [21] analyzed the impact of harvest time, malaxation duration and malaxation temperature on the concentration of specific phenols in EVOO prepared in a lab-scale olive mill from different varieties in Greece. Authors observed different trends for the concentration of various phenols (including oleocanthal) during the malaxation process and at different malaxation temperatures. Of note, both oleocanthal and oleacein increased their concentration in parallel with the increase in malaxation temperature, indicating that enzymatic activity favors the formation of these individual phenols at higher temperatures [21]. 

Another important variable influencing the TPC of EVOO is the potential olive tree pest infestation, particularly from *Bactrocera oleae* (the olive fruit fly) [103]. This insect still has the ability to affect an entire harvest if the climatic conditions of the year are particularly favorable to its spread, and its attack on the olive drupes frequently causes serious quantitative and qualitative damage to the milled product. Notario et al. [104] conducted a study to examine the changes induced by *Bactrocera oleae* infestation in the biosynthesis of volatile and phenolic compounds in olives (cvs. Picual, Manzanilla, and Hojiblanca). Oils obtained from infested fruits exhibited a significant increase in the content of some volatile compounds such as (E)-hex-2-enal, ethyl acetate, ethanol, and β-ocimene, along with a marked decrease in the phenolic contents. Authors also examined the impact of the changes induced by the attack of *Bactrocera oleae* on the expression of some genes that play a key role in the biosynthesis of volatile and phenolic compounds, such as lipoxygenase, polyphenol oxidase and β-glucosidase. The substantial reduction of phenolic content in the oils obtained from infested fruits was explained through the strong induction of a new olive polyphenol oxidase gene (*OePPO2*), suggesting the existence of a polyphenol oxidase (PPO)-mediated oxidative defense mechanism in olives [104].

Taking into account the aforementioned factors, which often preclude the possibility of complete control over the olive drupe ripening processes, we have shifted our attention to the variability of EVOO content of the polyphenol oleocanthal based on different cultivars and production methodologies. Monitoring the variations in oleocanthal levels in different monovarietal oils was the objective of scientific work conducted on Italian cultivars. Since there is a great variability in the phenolic composition of EVOOs due to origin, production technique and genotype, Negro et al. [105] conducted a study to assess the different phenolic profiles and antioxidant activity of monovarietal EVOOs. Authors confirmed the existence of such variability, as the overall content of oleuropein varied up to four times between eight distinct genotypes (from 33.80 to 152.32 mg/kg oil), whereas oleocanthal content was significant only in two olive oils. The antioxidant activity of olive oils was determined through 2,2-Diphenyl-1-picrylhydrazyl (DPPH) and oxygen radical absorbance capacity (ORAC) assays and was correlated with the content of total phenolic substances. IC_50_ values for the DPPH test ranged from 160 to 91 mg of oil, whereas the ORAC test showed values ranging between 5.45 and 8.03 μmol Trolox equivalent (TE)/g oil [105]. These results confirmed a high degree of variability in the content of phenolic compounds among EVOOs and attested that the antioxidant activity and nutraceutical value of EVOOs also depend on the biodiversity that characterizes the olive groves of the Mediterranean Region, especially those from the Italian territory. 

In the production of high-quality EVOOs, olive storage conditions should also be taken into account. Indeed, in a study conducted by Hachicha Hbaieb et al. [106], contents of secoiridoid compounds—assessed through high-resolution mass spectrometry—and their oxygenated, deacetoxylated and deacetoxy-oxygenated derivatives differed between the cultivars based on olive ripening degree and storage conditions. Such differences have been suggested to be caused by β-glucosidase, peroxydase and polyphenol oxidase activity changes during olive ripening and storage [106]. 

### 7.2. Oleocanthal-Rich EVOO Obtained from Olives Harvested in the Southern Italy “Cilento, Vallo di Diano and Alburni National Park” of the Campania Region (Province of Salerno)

In keeping with the abovementioned evidence, we have hereby reported our personal experience in the production of a certified organic EVOO (Lavanghe^®^) with a “Protected Designation of Origin” (PDO). This EVOO was obtained from olives of three different cultivars (*Rotondella*, *Frantoio*, and *Leccino*) harvested within the Southern Italy “Cilento, Vallo di Diano and Alburni National Park” of the Campania Region (Province of Salerno, Italy). These cultivars are included in the Cilento PDO production regulation. In this regard, it is important to highlight that the olive tree has found an ideal environment in the Campania Region thanks to the Mediterranean climate and the volcanic soils of this geographical area [107]. In the Campania Region, more than 85,000 olive oil farms spread over almost 75,000 hectares of utilized agricultural area, corresponding to almost 8% of the Italian olive oil utilized agricultural area. In particular, the Province of Salerno includes more than 38,000 olive farms thanks to ideal olive growing conditions determined by the climate and by the peculiar soils of its territory (including the “Cilento, Vallo di Diano and Alburni National Park”) [107].

In our experience, olives from *Olea europaea* L. plants of the *Rotondella*, *Frantoio* and *Leccino* cultivars were harvested at the beginning of maturation (so-called “first veraison”) and pressed within 6 h of harvest. These olive drupes came from olive trees at different heights in geographical areas located a short distance from one another (39.1 km) within the same National Park (villages of Gorga and Camella). Harvesting was organized gradually over 15 days (in the month of October 2020), keeping the harvesting criteria and extraction times stable for each day of harvest. The objective was to carry out the milling in the period of greatest theoretical polyphenol content within fresh olives, so they could be processed in the shortest time possible after harvesting. Table 2 summarizes the analytical report of the saponifiable and unsaponifiable fractions of the aforementioned certified organic EVOO (Lavanghe^®^), with the total polyphenol content (TPC) expressed as tyrosol complex. The aforementioned EVOO analytical report was issued by Chemiservice Srl (Monopoli, Italy), a laboratory specialized in chemical and microbiological analysis in the food, environmental, industrial and cosmetic sectors [laboratory recognized by the Italian Accreditation Body for Testing Laboratories ACCREDIA (appointed by the Italian government) and by the IOC (International Olive Council) for the performance of laboratory tests on olive oil and olive pomace oil]. The report shows the phenylethanoids (hydroxytyrosol and tyrosol) and the secoiridoids oleuropein aglycone, ligstroside aglycone and oleocanthal (indicated as “Decarboxymethyl ligstroside aglycone in open dialdehyde form”). As highlighted by the analytical report results, the detected concentrations of polyphenols reached substantially high values. Of note, the EVOO’s TPC amounted to 677 mg/kg, which is far above the usual EVOO’s concentration of phenolic compounds, that generally ranges between 100 and 300 mg/kg [18,98]. The high detected concentrations of hydroxytyrosol and derivatives (total polyphenols expressed as tyrosol complex) are also of notable interest, since such molecules are traditionally considered for defining the nutraceutical score based on the EFSA regulation. In this case, the concentration of total polyphenols (677 mg/kg, expressed as tyrosol complex) fully meets the EFSA requirement of 5 mg of hydroxytyrosol and its derivatives (e.g., oleuropein complex and tyrosol) per 20 g of olive oil [8,9]. In particular, 5 mg of hydroxytyrosol and its derivatives (e.g., oleuropein complex and tyrosol) per 20 g of olive oil corresponds to 250 mg of hydroxytyrosol and its derivatives (e.g., oleuropein complex and tyrosol) per kg of EVOO, a polyphenol concentration more than two times lower than the TPC found in the aforementioned analyzed polyphenol-rich EVOO (677 mg/kg). 

With regard to oleocanthal, this molecule usually represents about 10% of the total EVOO phenolic compounds (corresponding to about 10–30 mg/kg of EVOO) [18,98]. In the aforementioned analyzed EVOO, we detected a high oleocanthal concentration amounting to 141 mg per kg of EVOO [corresponding to 20.8% of the total EVOO phenolic compounds (677 mg/kg)]. It is important to note that, in the context of a regular intake of EVOO (25–50 mL), an oil like the one in question would provide between 3.23 and 6.46 mg of oleocanthal for each oil portion. Such oleocanthal concentrations are substantially higher than those previously estimated at 0.9 mg per 25–50 mL of EVOO [24,98]. These concentrations were calculated considering that one liter of oil weighs approximately 920 g, since the density of olive oil is 0.917 kg/L at 20 °C [108]. Therefore, 141 mg of oleocanthal per kg of EVOO is equivalent to 141 mg of oleocanthal per 1.09 L of EVOO.

Given the numerous and promising conclusions on the therapeutic potential of oleocanthal, the lack of specific reference for this polyphenol in the current legislation may be outdated. Indeed, the oleocanthal content of EVOO should be specified together with the mentioned “Hydroxytyrosol” and “Oleuropein Complex”. Currently, there is a large scientific consensus on the fact that the beneficial potential of EVOO on human health is due to the synergistic actions of its different fractions (saponifiable fraction and unsaponifiable fraction), and the ever-increasing number of studies on the anti-inflammatory and antioxidant properties of oleocanthal could soon push for greater recognition of the therapeutic potential of this molecule. However, the positive long-term effects of the regular consumption of nutraceutical EVOO have not been completely assessed yet. This could be partly due to an approach that has often overlooked the single EVOO compounds’ potential. Nevertheless, it is also worth noting that it has only been a few years since studies on polyphenol-rich olive oils or oleocanthal-rich olive oils have been conducted. If, in the past, olive oil was mostly extracted with rudimentary methods that could scarcely preserve the freshness of the olive fruit and, consequently, the active phenolic compounds, nowadays it is indeed possible to standardize a step-by-step production method that allows us to maximize the final yield of beneficial compounds, including selected polyphenols like oleocanthal. In terms of environmental sustainability, novel EVOO extraction methods (e.g., two-phase extraction process) also aim to reduce the negative environmental effects arising from the production of this product, such as those related to the agricultural phase and waste management [109]. Notably, Proietti et al. conducted a study aimed at evaluating the carbon footprint associated with the production of one liter of EVOO (chosen as a functional unit) through a life-cycle assessment approach to improve the environmental sustainability of the olive oil chain [110]. The studied olive groves were located in PDO areas of Umbria, a region of Central Italy. Authors concluded that: (a) olive groves are agricultural systems able to counteract climate change by mitigating the carbon dioxide emissions into the atmosphere associated with the oil production chain; and (b) EVOO can represent a potential carbon-negative product under specific conditions, mainly if it is of local origin (“zero-km-product”) and if it is produced through traditional practices [110].

## 8. Discussion 

Research applied to food and its effect on human health and diseases has undoubtedly seen a sustained boost in recent decades, particularly because verifying previous evidence using new methodologies has made it possible to identify an ever-increasing number of new metabolites in known functional foods. In this context, a few examples can be used to describe this trend in scientific research, such as that of EVOO, a plant product that has been processed for thousands of years and whose properties have often been mistakenly believed to be sufficiently known. The progressive investigations, starting from the studies of the American physiologist Ancel Benjamin Keys [111], have somehow segmented the research work, evaluating the effects on human health of the two main fractions of olive oil, the saponifiable fraction and the unsaponifiable fraction. Given the assumption that obesity and heart disease rates negatively affected life expectancy, the Mediterranean diet was identified by Keys as a primary characteristic among groups of individuals who were largely immune to these trends. Following research on the impact of food quality on human health, in 1958 Keys launched the first major epidemiological longitudinal study of dietary habits across different countries and their effects on heart disease and longevity (known as the “Seven Countries Study”) [111]. In an article containing personal reflections on the Mediterranean diet, Keys stated that his concern about diet as a public health problem began in the early 1950s in the Italian city of Naples (located in the Campania Region), where there was a very low incidence of coronary heart disease associated with the so-called “good Mediterranean diet,” whose composition was described as “mainly vegetarian: pasta in many forms, leaves sprinkled with olive oil, all kinds of vegetables in season, and often cheese, all finished off with fruit, and frequently washed down with wine” [112]. In 2010, the Mediterranean diet was awarded the UNESCO (United Nations Educational, Scientific and Cultural Organization) recognition as an Intangible Cultural Heritage of Humanity due to its complex interplay between distinct factors, including knowledge, skills, processing, cooking and especially the sharing and consumption of food [113]. Since then, several studies have confirmed the human health benefits of EVOO [7,114], which is one of the most important ingredients of the Mediterranean diet that is consistently consumed as the main source of fat across Mediterranean countries [115]. Current research is being focused on deciphering the exact effects of EVOO’s bioactive compounds (including polyphenols) on processes such as inflammation, coagulation, platelet aggregation, fibrinolysis, oxidative stress, endothelial function and lipid homeostasis, whose dysregulation can contribute to the development of several chronic non-communicable diseases (including cancers and neurodegenerative disorders) [115,116].

The saponifiable fraction of EVOO mainly expresses the nutraceutical value of the well-known monounsaturated omega-9 fatty acid oleic acid, while current knowledge of the unsaponifiable fraction has shed new light on the promising activities of an entire family of molecules such as the minor polar compounds. For this last large group of bioactive molecules, research has long hypothesized an indiscriminate beneficial effect on human health as it was first considered a simple grouping of natural antioxidants. Indeed, the literature on olive oil polyphenols has traditionally maintained this character of investigation “by group,” essentially testing the collective potential of such molecules, as a group, on cardiometabolic parameters (e.g., lipid profile and endothelial dysfunction). The most evident mechanism of action on cellular free radicals was thus considered to be the main factor responsible for the biological activity of polyphenolic compounds. Yet, subsequent evidence of polyphenols’ efficacy against different inflammatory diseases gradually suggested that the mechanisms of action involved were multiple and very distinct from the single antioxidant capacity [117].

Over the last few years, evidence kept accumulating regarding the selective activities of specific compounds such as hydroxytyrosol, tyrosol and oleuropein, and it laid the foundations for regulating the optional information on the label of olive oil products. This is also reflected in the previously used terminology, which aggregated these molecules mentioned under “Hydroxytyrosol and derivatives”. Oleocanthal ultimately represents an excellent example of the relatively new molecules tested in EVOO. What can be observed when consulting the scientific evidence is indeed a marked change of pace around the first decade of the 2000s, when the studies on polyphenols contained in EVOOs of different geographical origins substantially moved from general investigations of the polyphenol molecule class to more detailed investigations of individual compounds such as tyrosol, hydroxytyrosol, oleacein, oleuropein and oleocanthal, among others. Over the last decades, the shift of research focus towards the investigation of individual EVOO compounds has certainly favored a better understanding of the peculiar biologic actions of different polyphenols. In this regard, secoiridoids—like oleocanthal—have demonstrated an interesting joint activity on human diseases. It is likely that by continuing on this research path, new molecules will be identified, allowing for a better understanding of the pleiotropic human health benefits of different secoiridoids [118]. Intriguingly, a study analyzing the phenolic profile of Italian monovarietal and multivarietal EVOO samples (including PDO oils) from different Italian regions (Sicily, Puglia, Lazio and Tuscany) through high-performance liquid chromatography—using photodiode arrays and mass spectrometry detectors—found three unknown compounds that were tentatively attributed to the phenolic compounds class based on their ultraviolet (UV) spectra [119]. Furthermore, if we include in the reasoning the concomitant attention of nutrition research for lignans and α-tocopherol (vitamin E) contained in EVOO, we can quickly realize how the EVOO therapeutic potential is even wider than previously thought.

These remarks represent the key to understanding the rationale for our literature review, since this innovative way of considering EVOOs represents the main difference from the past. Until recently, what was considered the exclusive trait of new good agricultural practices actually had even more important implications for human health. The traditional consumption of EVOO by Mediterranean people documented in early observational studies and its effects on human health should be further investigated in studies examining the health effects of EVOOs obtained through novel methodologies aimed at increasing the final content of bioactive molecules (like oleocanthal). In fact, if we talk about the ancestral past of this food, we are referring to a plant extract that often originated from olives left to fall on the ground at an advanced stage of maturation, worsening the fermentation processes already established in the olive drupe due to uncontrolled insect pest infestation. Such olives underwent milling in the open environments of ancient oil mills, with the olive paste exposed to the atmosphere’s oxidative action and uncontrolled temperatures during malaxation. Although the evidence on the health effects of the habitual consumption of such a food product was still visible and measurable at that time, this now only attests to the enormous but still unexpressed therapeutic potential for nutraceutical EVOOs. Molecules such as oleocanthal are important indicators of this therapeutic potential, and their high concentrations in the finished food product (EVOO) are strictly correlated with the time and methods of harvesting and processing of fresh olives, with the inevitable variability found between the different cultivars coming from numerous areas of the Mediterranean Region.

New technologies will also undoubtedly represent a precious ally for controlling the health of the olive tree in territories where the marked climate variability and socioeconomic transformations expose olive crops to ever-increasing biosafety risks, such as infestation by newly emerging pests. Novel and sustainable olive crop management and protection methods may provide olive farmers with the required capacity to respond to present and future agricultural challenges. For instance, tree endo-therapy is a precision agriculture method consisting of systemic delivery of active ingredients into the plant vascular system performed in a precise manner via physical trunk injections, without risks of off-target drifts [120]. Innovative formulation technologies allowing for controlled release of active ingredients represent another promising tool for more targeted and sustainable olive crop management and protection [120]. Furthermore, the EVOO extraction process assisted through novel and emerging technologies (e.g., pulsed electric fields, high-pressure processing, high-power ultrasound and microwave heating technologies) has proven to be very efficient on olive paste, resulting in a significant increase in the oil yield [121]. With regard to the content of bioactive compounds (such as phenols, phytosterols and vitamin E, among others), the oil quality parameters (oxidative stability and peroxide value) and sensory attributes are improved after the extraction process assisted through such emerging technologies [121]. 

In view of the above, the emerging recognition of oleocanthal as a potential antineoplastic and neuroprotective agent warrants a future expansion of label indications required by EFSA to claim that an EVOO has nutraceutical properties. In particular, EVOO’s nutrition facts label should contain information on the content of specific polyphenols (including oleocanthal) in order to increase consumer awareness about EVOO composition and its related implications for human health. Italian EVOO production accounts for approximately 15% of the global EVOO market, and population surveys indicate that Italian consumers are attracted to the high EVOO quality since they appreciate the recognized human health benefits of this food product, as well as its unique organoleptic properties [122]. Possible strategies aimed to spread the scientific knowledge of EVOO’s health benefits and to raise people awareness about EVOO quality choices are currently under investigation [123].

With specific regard to oleocanthal, the oral pungency sensation evoked by EVOO ingestion is currently one of the few organoleptic characteristics of olive oil that consumers can empirically exploit to identify oleocanthal-rich EVOOs. Therefore, based on the current evidence for human health benefits of oleocanthal and other EVOO polyphenols, the update of regulation on EVOO’s nutrition facts label will certainly give greater prominence to such polyphenols (including oleocanthal) as bioactive metabolites. 

Regarding the relationship between oleocanthal chemical structure and oleocanthal biologic activities, the two aldehyde groups of oleocanthal have been proven to be essential for the prevention of tau fibrillogenesis [74]. However, the structure-activity relationship of oleocanthal and other EVOO polyphenols needs to be further investigated in future studies that may pave the way for the development of novel anticancer and neuroprotective drugs starting from the molecular scaffolds of such naturally occurring compounds [124]. 

The translation of the promising findings regarding oleocanthal use in animal models into clinical applications remains difficult for a number of reasons, such as the interspecies differences in pharmacokinetics and pharmacodynamics [24]. On the other hand, it is commonly believed that the oleocanthal doses associated with health benefits in animal studies (between 5 mg and 30 mg/kg/day) [24] may not be physiologically achievable in humans through olive oil intake, since the reasonable daily intake of 25–50 mL of EVOO is thought to provide not more than 0.9 mg of oleocanthal, as it has previously been postulated [24,98]. Yet, this issue can be easily overcome nowadays thanks to the current availability of oleocanthal-rich EVOOs, whose consumption has proven to be safe and associated with beneficial health effects in preliminary clinical studies [53].

In order to provide a better understanding of the composition of an oleocanthal-rich EVOO, in this review we also reported the example of an analytical report of a certified oleocanthal-rich EVOO with a protected designation of origin resulting from laboratory tests and from the judgment of a panel of certified experts, which currently constitutes the best guarantee of compliance with rigorous quality parameters. The values shown in this report regard a Mediterranean (Italian) EVOO from Southern Italy’s “Cilento, Vallo di Diano and Alburni National Park” of the Campania Region (Province of Salerno, Italy) and result from our direct experience in the advanced techniques of olive oil production employed today, which allow for the production of polyphenol- and oleocanthal-rich EVOOs with a high nutraceutical value.

## 9. Conclusions

In conclusion, particularly in the field of plant-based foods consumed as health supplements, the potential of beneficial bioactive molecules, like oleocanthal, is unequivocally and closely linked with the appropriateness of agricultural procedures during the production of the food (e.g., EVOO), either fresh or processed. The current evidence on anticancer and neuroprotective effects of oleocanthal mainly derives from in vitro and animal studies. Therefore, large prospective clinical studies are needed to confirm the beneficial effects of oleocanthal in humans, particularly at physiologically achievable doses. Such studies may be based on the dietary consumption of oleocanthal-rich EVOOs and/or on the use of pure oleocanthal administered as a supplement. The safety profile of oleocanthal treatment (at different doses) also needs to be confirmed in clinical studies, although preclinical studies investigating the oleocanthal anticancer properties showed that this molecule exerts selective antiproliferative effects on cancer cells without influencing the cell cycle of normal cells. Another important aspect that needs to be ascertained is whether oleocanthal can effectively exert synergistic effects with conventional anticancer drugs in clinical settings, as it has been suggested in preclinical studies. Finally, mechanistic studies are also warranted in order to better define the exact biologic activities of oleocanthal in humans, as well as its pharmacokinetics, pharmacodynamics and dose-response relationship in different clinical settings. 

## Figures and Tables

**Table 1:** Subclasses of the main minor polar compounds found in extra virgin olive oil (EVOO).

Subclass	Molecule
**Secoiridoids**	(a) Oleuropein aglycone (b) Deacetoxy-oleuropein aglycone (c) Oleocanthal (d) Oleacein (e) Ligstroside aglycone
**Phenylethanoids**	(a) Hydroxytyrosol (b) Tyrosol
**Phenolic acids**	(a) Gallic acid (b) Protocatechuic acid (c) p-Coumaric acid (d) Ferulic acid (e) p-Hydroxybenzoic acid (f) Vanillic acid (g) Caffeic acid (h) Syringic acid (i) Cinnamic acid
**Lignans**	(a) (+)-Pinoresinol (b) (+)-1-Acetoxypinoresinol
**Flavonoids**	(a) Luteolin (b) Apigenin

**Table 2:** Analytical report of the saponifiable and unsaponifiable fractions of a certified organic polyphenol- and oleocanthal-rich extra virgin olive oil (EVOO) with a “Protected Designation of Origin” (PDO) and obtained from olives of three different cultivars (*Rotondella*, *Frantoio,* and *Leccino*) harvested in geographical areas located at a short distance from one another (villages’ name: Gorga and Camella) within the Southern Italy “Cilento, Vallo di Diano and Alburni National Park” of the Campania Region (Province of Salerno, Italy). The method listed as “NGD C89-2010” refers to the analysis and characterization of EVOO’s polyphenols performed through high-performance liquid chromatography (HPLC). Abbreviations: meq O_2_, milliequivalents of active oxygen. * With regard to EVOO polyphenols, an established limit is only available for total polyphenols (expressed as tyrosol complex).

	Value	Unit of Measurement	Method	Limit
**Organoleptic characteristics**				
**Aspect**	Limpid			
**Color**	From green to straw yellow			
**Odor**	Fruity (medium)			
**Flavor**	Fruity, with medium bitter and spicy sensation			
**Defects**	Absent			
**Free fatty acids**	0.20	Percentage (%) of oleic acid	According to Commission Regulation (EEC) No. 2568/91 on the characteristics of olive oil and olive pomace oil and on the relevant methods of analysis	0.7
**Peroxide value**	7.7	meq O_2_/kg oil	According to Commission Regulation (EEC) No. 2568/91 on the characteristics of olive oil and olive pomace oil and on the relevant methods of analysis	12
**Biophenols ** **(Polyphenols)**				
**Total** **Polyphenols** **(expressed as tyrosol complex)**	677	mg/kg	NGD C89-2010	≥80 *
**Hydroxytyrosol (3,4 DHPEA)**	14	mg/kg	NGD C89-2010	*
**Tyrosol (p, HPEA)**	11	mg/kg	NGD C89-2010	*
**Decarboxy** **methyl-oleuropein aglycone in open dialdehyde form** **(3,4 DHPEA–EDA)**	147	mg/kg	NGD C89-2010	*
**Decarboxy** **methyl-ligstroside aglycone in open dialdehyde form (p, HPEA-EDA)**	141	mg/kg	NGD C89-2010	*
**Lignans**	72	mg/kg	NGD C89-2010	*
**Oleuropein aglycone** **(3,4 DHPEA-EA)**	107	mg/kg	NGD C89-2010	*
**Ligstroside aglycone** **(p, HPEA-EA)**	31	mg/kg	NGD C89-2010	*

## Data Availability

The original analytical report of the certified organic polyphenol- and oleocanthal-rich extra virgin olive oil (Lavanghe^®^ EVOO) summarized in Table 2 is not included in this manuscript but is available from the corresponding author (M.I.) upon reasonable request (original analytical report in Italian). The EVOO analytical report and quality certification were issued by Chemiservice Srl (Monopoli, Italy), a laboratory specialized in chemical and microbiological analysis in the food, environmental, industrial and cosmetic sectors [laboratory recognized by the Italian Accreditation Body for Testing Laboratories ACCREDIA—appointed by the Italian government—and by the IOC (International Olive Council) for the performance of laboratory tests on olive oil and olive pomace oil; URL: www.chemiservice.com/en/—accessed on 29 October 2023.
